# Reintervention using a through-the-mesh technique within the abdominal cavity following stent migration after endoscopic ultrasound-guided hepaticogastrostomy

**DOI:** 10.1055/a-2491-1094

**Published:** 2024-12-12

**Authors:** Takeshi Ogura, Yuki Uba, Takafumi Kanadani, Kimi Bessho, Hiroki Nishikawa

**Affiliations:** 1 Endoscopy Center, Osaka Medical and Pharmaceutical University Hospital, Takatsuki, Japan; 2 2nd Department of Internal Medicine, Osaka Medical and Pharmaceutical University, Takatsuki, Japan

Endoscopic ultrasound-guided hepaticogastrostomy (EUS-HGS) is now widely performed in patients for whom endoscopic retrograde cholangiopancreatography is contraindicated, such as patients with duodenal obstruction or surgically altered anatomy 1 2 3 . Although EUS-HGS has a clinical effect in selected patients, the rate of adverse events is not very low. In addition, critical adverse events such as stent migration into the abdominal cavity have been reported, although various efforts to prevent stent migration have also been proposed 4 5 . If stent migration occurs, surgical treatment is normally considered. However, EUS-HGS itself is usually performed for patients with advanced malignant tumors; therefore, surgical treatment might not be appropriate. Here we describe technical tips for successful reintervention using a through-the-mesh technique via the abdominal cavity following stent migration after EUS-HGS.

An 80-year-old woman was admitted to our hospital with obstructive jaundice due to pancreatic cancer. As duodenoscope insertion into the second part of the duodenum failed due to malignant duodenal obstruction, EUS-HGS was attempted. A partially covered self-expandable metal stent (HANARO Benefit; M.I Tech., Seoul, South Korea) was successfully deployed from the intrahepatic bile duct to the stomach without any adverse events ( Fig. 1 ). However, 7 days later, the patient showed increased inflammatory markers and had abdominal pain. On computed tomography, stent migration was diagnosed. Reintervention was therefore attempted.

**Fig. 1:**
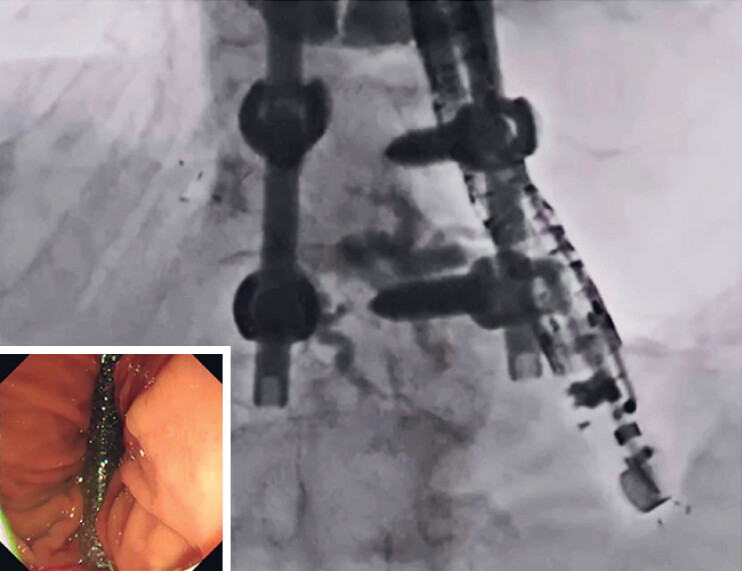
A partially covered self-expandable metal stent was successfully deployed.

First, the stomach was carefully checked, but the stent was not found. Therefore, an EUS scope was inserted, the EUS-HGS stent was identified, and the stent was punctured using a 19-G needle ( Fig. 2 ), with successful puncture confirmed by visualization of contrast medium ( Fig. 3 **a** ). After guidewire deployment, an uneven catheter was inserted into the stent, and double guidewires were deployed to improve scope stability and ensure a safe procedure ( Fig. 3 **b** ). Finally, the stent delivery system was successfully inserted and deployed from the stent to the stomach ( Fig. 3 **c** , Video 1 ).

**Fig. 2:**
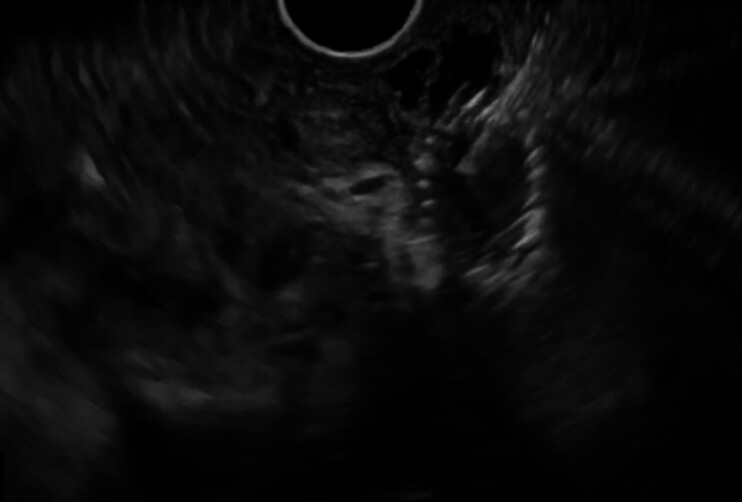
The stent placed for endoscopic ultrasound-guided hepaticogastrostomy was punctured using a 19-G needle.

**Fig. 3:**
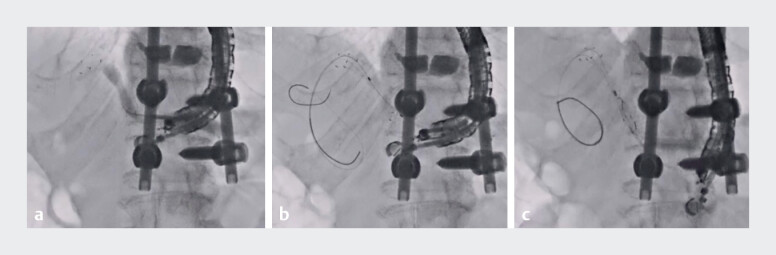
Placement of the rescue stent. **a** Successful stent puncture was confirmed by visualization of contrast medium. **b** After guidewire deployment, an uneven catheter was inserted into the stent, and double guidewires were deployed to improve scope stability and ensure a safe procedure. **c** The stent delivery system was successfully inserted and deployed from the stent to the stomach.

The endoscopic ultrasound-guided hepaticogastrostomy stent was punctured using a 19-G needle.Video 1

The patient’s clinical condition improved, and 5 days later, she was discharged. During the 2-month clinical follow-up, no stent migration was observed.

Biliary access through the proximal hole of the EUS-HGS stent has been reported as an alternative technique for treating a migrated EUS-HGS stent 5 . This salvage technique is useful, but detection of the proximal hole in the EUS-HGS stent is usually only successful if the migrated stent is optimally orientated. In addition, the pushing force applied might not be effectively transmitted because of the long distance between the migrated EUS-HGS stent and the biliary tract. Compared with this technique, the present technique has several advantages such as easy detection of the migrated EUS-HGS stent and favorable push ability because the distance between the migrated EUS-HGS stent and the biliary tract is short.

In conclusion, the present technique can provide a new rescue approach after stent migration and might avoid surgical treatment.

Endoscopy_UCTN_Code_TTT_1AS_2AG

## Notes

Endoscopy E-Videos https://eref.thieme.de/e-videos
*E-Videos* is an open access online section of the journal *Endoscopy* , reporting on interesting cases and new techniques in gastroenterological endoscopy. All papers include a high-quality video and are published with a Creative Commons CC-BY license. Endoscopy E-Videos qualify for HINARI discounts and waivers and eligibility is automatically checked during the submission process. We grant 100% waivers to articles whose corresponding authors are based in Group A countries and 50% waivers to those who are based in Group B countries as classified by Research4Life (see: https://www.research4life.org/access/eligibility/ ). This section has its own submission website at https://mc.manuscriptcentral.com/e-videos .
